# Companion Tumor Sequencing to Assess the Clinical Significance of Germline Sequencing in Children With Cancer

**DOI:** 10.1001/jamanetworkopen.2021.35135

**Published:** 2021-11-18

**Authors:** Tinaye Mutetwa, Catherine Goudie, William D. Foulkes, Paz Polak

**Affiliations:** 1 Department of Oncological Sciences, Icahn School of Medicine at Mount Sinai, New York City, New York; 2 Research Institute, McGill University Health Centre, Montreal, Quebec, Canada; 3 Department of Human Genetics, McGill University, Montreal, Quebec, Canada; 4 Tisch Cancer Institute, Icahn School of Medicine at Mount Sinai, New York City, New York

## Abstract

This case-control study assesses the clinical significance of germline sequencing in children with cancer.

## Introduction

The increasing uptake of multigene sequencing of germline DNA from children with cancer has led to an increase in the discovery of high- and moderate-penetrance germline pathogenic or likely pathogenic (P/LP) variants (here, combined as high/moderate-penetrance germline pathogenic variants, or hmGPVs) in persons with cancer.^1^ Some of these hmGPVs are truly associated with the cancer, whereas others are not. We set out to quantify the etiological association between hmGPVs in associated and nonassociated pediatric and adult-onset cancers.

## Methods

This case-control study used publicly available deidentified human data and does not require institutional review board approval per the Tri-Council Policy Statement “Ethical Conduct for Research Involving Humans” Article 2.2b (TCPS2, 2018) and the Icahn School of Medicine at Mount Sinai (ISMMS) Human Subjects Research (HSR) Determination Guidance. We used tumor zygosity of hmGPVs in tumor suppressor genes (TSG) to ascertain whether hmGPVs are associated with tumorigenesis. We used germline and zygosity data from 2 recent prospectively matched tumor-normal panel-based germline testing studies: IMPACT in 751 pediatric patients with solid tumors (n = 99 hmGPVs)^2^ and Mi-ONCOSEQ in 1015 adult metastatic tumors (n = 129 hmGPVs).^3^ Zygosity of hmGPVs was determined using loss of heterozygosity (LOH) or a second coding sequence somatic hit. We determined if tumors were associated or nonassociated (eMethods in the Supplement). For the analysis of the pediatric data set, there were 45 eligible hmGPVs in associated tumors and 18 in nonassociated tumors. Among adult cancers, 59 and 55 hmGPVs were in associated and nonassociated tumors, respectively. The primary outcome was percentage of patients with GPVs with second hits in associated and nonassociated tumors. Two-sided χ^2^ test was used to determine proportion differences. The threshold for statistical significance was set at *P* < .05. We used R statistical software, version 4.0.5 (R Project for Statistical Computing).

## Results

A total of 99 pediatric patients (49 [49%] female; median age, 5 years [range, 0-19 years]), and 129 adult patients (65 [50%] female; median age, 56 years [range, 18-81 years]) with hmGPVs were included the analysis. Demographic data are shown in the Table. Forty-one of 45 pediatric tumors (91%) with an hmGPV in a TSG with zygosity information had LOH or a second somatic hit within the coding region of the gene (Figure). In contrast, only 1 of 18 (5.5%) hmGPVs in nonassociated tumors had a second hit in the tumor (difference, 85.6%; 95% CI, 62.1%-92.6%; *P* < .001) (Figure).^2^

**Table.** Demographics of Patients With High- and Moderate-Penetrance Germline Pathogenic Variants

Characteristic	No. (%)	
	Pediatric tumors, IMPACT Study, Fiala, EM, et al (n = 99)	Adult tumors, Mi-ONCOSEQ Study, Cobain, EF, et al (n = 129)
Age, median (range), y	5 (0-19)	56 (18-81)
Gender		
Male	50 (51)	64 (50)
Female	49 (49)	65 (50)
Race and ethnicity		
African American or Black	7 (7)	NA
Ashkenazi or Sephardic Jewish	15 (15)	NA
Hispanic	9 (9)	NA
Asian	11 (11)	NA
European	32 (32)	NA
Yemeni	1 (1)	NA
No data	24 (24)	NA
Tumor association with hmGPVs		
Maybe	11 (11)	2 (2)
Associated	60 (61)	70 (54)
Not associated	28 (28)	57 (44)
Patients with hmGPV in tumor suppressor and known zygosity and association determined	63 (100)	114 (88)
Pediatric tumor category		
Central nervous system	21 (21)	NA
Neuroblastoma	14 (14)	NA
Retinoblastoma	26 (26)	NA
Sarcoma	20 (20)	NA
Other	18 (18)	NA
Adult tumor category		
Breast	NA	26 (20)
Ovary	NA	5 (4)
Prostate	NA	15 (12)
Colorectal cancer	NA	3 (2)
Lung	NA	2 (2)
Sarcoma	NA	17 (13)
Adrenocortical carcinoma	NA	3 (2)
Gastroesophageal	NA	6 (5)
Glioblastoma mutliforme	NA	4 (3)
Hepatopancreatobiliary	NA	12 (9)
Kidney/ureter/bladder	NA	8 (6)
Other carcinoma	NA	19 (15)
Other non-carcinoma malignancy	NA	9 (7)

Abbreviations: hmGPVs, high/moderate-penetrance germline pathogenic or likely pathogenic (P/LP) variants; NA, not available.

**Figure.**
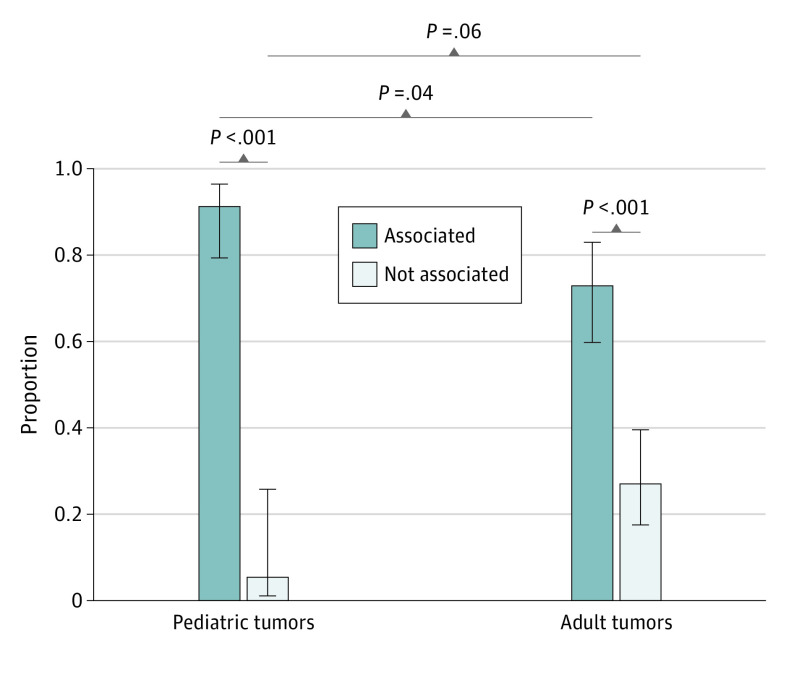
Zygosity Status of hmGPVs in Pediatric and Adult Tumors Depicted are high/moderate-penetrance (hm) germline pathogenic or likely pathogenic variants (here, combined as germline pathogenic variants [hmGPVs]) present in tumors associated or not associated with the altered genes for pediatric and adult tumors. The *P* values indicated were obtained from running comparisons of proportions tests. A decision on whether pediatric tumors were associated or not associated was determined by C.G. and W.D.F. For adult tumors, W.D.F. made the determinations alone. Please see the eMethods in the Supplement for more details.

Of 22 RB1-associated tumors with an *RB1* GPV that had available zygosity information, 20 had second hit. In contrast, none of 3 cases in non–RB1-associated tumor types had second hits (although the *RB1* GPVs likely played a role in the preceding bilateral retinoblastomas that were not tested). In contrast, 5 patients with *BRCA1*, *BRCA2*, and *PALB2*—all found in patients with tumor types not known to be associated with GPVs in these genes—did not have second hit and are unlikely to respond to PARP inhibitors.^4^

From the adult study, 16 of 59 (27%) nonassociated tumors with an hmGPV harbored second hits compared with 40 of 55 (73%) in patients with associated tumors (difference of 45.6%; 95% CI, 27.6%-59.5%; *P* < .001) (Figure).^3^

## Discussion

The high frequency of biallelic hits observed for TSGs that are known to be associated with the cancer in which the hmGPVs occurred indicate that in pediatric patients with cancer, when hmGPVs are detected in associated tumors, these variants are almost always associated with tumor development. In contrast, when the association between hmGPV and the cancer is not established, a hmGPV in a child with this class of cancer is rarely associated with the tested tumor. In adult carriers of hmGPVs, cancers can arise from multiple mechanisms, whereas in children, exposures are limited.^5^ These results demonstrate how companion tumor sequencing can be informative in determining the etiologic role of germline variants,^6^ especially during broad germline testing beyond associated cancer types for both adult and pediatric cases. This study has several limitations, such as a small sample size, especially regarding specific pairs of tumors and genes. In addition, functional assays to determine the role of hmGPVs are needed in addition to information on zygosity.

## Notes

Supplement. **eAppendix. **Determination of the association status of pediatric and adult tumors
eReferences.
