# Supplementary material for: Companion Tumor Sequencing to Assess the Clinical Significance of Germline Sequencing in Children With Cancer
Source: JAMA Netw Open. 2021 Nov 18;4(11):e2135135. doi: 10.1001/jamanetworkopen.2021.35135 (PMC8603075; doi:10.1001/jamanetworkopen.2021.35135)
Supplement: Supplement. — eAppendix. Determination of the association status of pediatric and adult tumors eReferences. [file jamanetwopen-e2135135-s001.pdf]

Supplemental Online Content

Mutetwa T, Goudie C, Foulkes WD, Polak P. Companion tumor sequencing to assess the clinical significance of germline sequencing in children with cancer. *JAMA Netw Open*. 2021;4(11):e2135135. doi:10.1001/jamanetworkopen.2021.35135

eAppendix. Determination of the association status of pediatric and adult tumors

eReferences

This supplemental material has been provided by the authors to give readers additional information about their work.

© 2021 Mutetwa T et al. *JAMA Network Open.*

eAppendix. Determination of the association status of pediatric and adult tumors

We used germline and zygosity data from two recent prospectively matched tumor-normal panel-based germline testing studies: for childhood cancer, we used IMPACT(clinicaltrials.gov NCT01775072),[1] and for adults, Mi-ONCOSEQ.[2] Whether pediatric tumors were associated or not associated was determined by CG and WDF. This determination was based on the extensive literature review that formed the basis of the construction of the algorithms in a cancer predisposition screening tool (www.MIPOGG.com). This tool has been described in several publications[3-6] . If there was no agreement, we assumed the tumor was associated. In cases where neither CG nor WDF were able to be decisive (n = 13), these cases were excluded. For adult tumors, WDF made the determinations alone, based on clinical experience, his publications[7-14], and the publications of others (not cited). For two cases, no decision was made, and these cases were not included in the analysis.

eReferences

1. Fiala EM, Jayakumaran G, Mauguen A, et al. Prospective pan-cancer germline testing using MSK-IMPACT informs clinical translation in 751 patients with pediatric solid tumors. *Nature Cancer* 2021.
2. Cobain EF, Wu YM, Vats P, et al. Assessment of Clinical Benefit of Integrative Genomic Profiling in Advanced Solid Tumors. *JAMA Oncol* 2021; **7**(4): 525-33.
3. Cullinan N, Schiller I, Di Giuseppe G, et al. Utility of a Cancer Predisposition Screening Tool for Predicting Subsequent Malignant Neoplasms in Childhood Cancer Survivors. *J Clin Oncol* 2021: JCO2100018.
4. Cullinan N, Villani A, Mourad S, et al. An eHealth decision-support tool to prioritize referral practices for genetic evaluation of patients with Wilms tumor. *Int J Cancer* 2020; **146**(4): 1010-7.
5. Goudie C, Cullinan N, Villani A, et al. Retrospective evaluation of a decision-support algorithm (MIPOGG) for genetic referrals for children with neuroblastic tumors. *Pediatr Blood Cancer* 2018; **65**(12): e27390.
6. Goudie C, Coltin H, Witkowski L, Mourad S, Malkin D, Foulkes WD. The McGill Interactive Pediatric OncoGenetic Guidelines: An approach to identifying pediatric oncology patients most likely to benefit from a genetic evaluation. *Pediatr Blood Cancer* 2017; **64**(8).
7. Foulkes WD. A tale of four syndromes: familial adenomatous polyposis, Gardner syndrome, attenuated APC and Turcot syndrome. *QJM* 1995; **88**(12): 853-63.
8. Foulkes WD, Polak P. Li-Fraumeni Syndrome in the Cancer Genomics Era. *J Natl Cancer Inst* 2021.
9. Sherman ME, Foulkes WD. BRCA1/2 and Endometrial Cancer Risk: Implications for Management. *J Natl Cancer Inst* 2021.

© 2021 Mutetwa T et al. *JAMA Network Open.*

10. Foulkes WD. The ten genes for breast (and ovarian) cancer susceptibility. *Nat Rev Clin Oncol* 2021; **18**(5): 259-60.
11. Hughley R, Karlic R, Joshi H, Turnbull C, Foulkes WD, Polak P. Etiologic Index - A Case-Only Measure of BRCA1/2-Associated Cancer Risk. *N Engl J Med* 2020; **383**(3): 286-8.
12. Rivera B, Polak P, Foulkes WD. Monogenic Diseases of DNA Repair. *N Engl J Med* 2018; **378**(5): 491.
13. Foulkes WD. Inherited susceptibility to common cancers. *NEnglJMed* 2008; **359**(20): 2143-53.
14. Hodgson SV, Foulkes WD., Eng C, Maher ER. A Practical Guide to Human Cancer Genetics. Switzerland: Springer Nature; 2014.

© 2021 Mutetwa T et al. *JAMA Network Open.*
